# On the Overlap of Systemic Events: Covid-19, Climate, and Journalism

**DOI:** 10.1177/2056305120948197

**Published:** 2020-08-11

**Authors:** Risto Kunelius

**Affiliations:** University of Helsinki, Finland

**Keywords:** Covid-19, climate change, expertise, events, journalism

## Abstract

Covid-19 represents a systemic event—a state of emergency—that disrupts the routines of societies from the level of individuals to institutions, nations, and global interaction. Revealing the vulnerability of the intensively interconnected world suggests a juxtaposition with another systemic crisis: the climate emergency. Drawing on some key literature on the different aspects of “events”—as heightened political semiosis (Wagner-Pacifi), as (possible) transformation of social and symbolic structures (Sewell), and as moments where new horizons are opened (Arendt)—this essay suggests three intersecting themes where reactions to Covid-19 help to sharpen the crucial questions of future journalism: the role of “knowledge” and expertise, the power of national framing, and the challenge of covering the new imperatives and possibilities of everyday life.

Events appear against the stable background of the usual and the expected, sometimes dramatically. Amitav Gosh’s (2016) reflection on climate change, The Great Transformation, starts with a surprise “extreme weather event”—an unseasonal cyclone in New Delhi in 1978. The storm nearly kills him. The memory spurs Gosh to consider the ability of non-human forces to intervene in human affairs, to disrupt our realist world horizon and cause an uncanny sense of uncertainty.

Events take shape and meaning against the taken-for-granted backdrop. Literary genre of realism exemplifies this dynamic well: detailed descriptions of the milieu, landscape, and people’s everyday habits set the stage for something worth telling (Gosh, 2016, pp. 1–84). By “routinizing the unexpected,” to borrow twist a phrase from Gaye Tuchman (1973), newsrooms have been a particularly effective institution of realism; there are always some “events” to report, yet at the same time the steady flow of news constructs the background against which the unexpected appear.

Covid-19 has turned out to be an exceptionally powerful, unfolding event. The virus turns the effectivity of an intensively networked and interdependent world into a potential vulnerability. It shakes the mutual expectations of individuals, established institutions, entire societies, and global systems. Such moments of systemic crisis makes us ask: What if our expectations about everyday reality—about weather, home, work, mobility, and so on—are no longer quite valid? What if the state of exception is not just a temporary suspension of the normal but actually signals the arrival of something yet unknown?

Making sense of such events while they are unfolding evokes associations, comparisons, and cross exposures. What will this mean for the questions we were preoccupied with before? Will the pandemic suppress voter turnout? Will it fuel efforts by major tech companies to consolidate their power (e.g., Klein, 2020)? Will it end in expanded surveillance (e.g., Agamben, 2020)? Will human and civil rights “recover” from states of emergency (Gozdecka, 2020)? How will constitutional order hold up (e.g., Grogan, 2020)? How might the pandemic exacerbate social inequality (e.g., Reich, 2020)?

What does a systemic event like Covid-19 mean for media and journalism (research)? A useful way to pose this question is to look at the overlapping challenges revealed by immediate reactions to the pandemic and imperatives of adjusting to the unfolding climate crisis. Drawing from some key literature on “events,” I will raise three concerns for future journalism: the role of expertise, the weight of the nation state, and the challenge of reporting changing everyday life. They point not only to the problematic legacy of journalism but also to new opportunities that these overlapping systemic states of “emergency” suggest.

## Expertise in Intense Political Semiosis

Robin Wagner-Pacifi (2018) sees an event as a process of intensified *political semiosis*, an unpredictable whirl of meanings, where three aspects of public speech and action intertwine. The *performative* power of speech creates and changes realities, causing tangible effects (e.g., a government declaration of a state of emergency). *Demonstrative* aspects of words and gestures signal and shape relationships in time, in space and between people (e.g., referring to coronavirus as the “Chinese virus).” Speech acts also function as *representation*, they craft interpretations for circulation (e.g., comparing the pandemic to a “war”) (Wagner-Pacifi, 2018, pp. 16–32).

The heightened political semiosis of events is unpredictable for all actors drawn into its orbit. But a particularly interesting role in the pandemic has fallen to experts, who have had to handle both an exceptional demand for information and the political need to justify exceptional acts of governance, like explicit suspension of civil rights. Despite this tension, national epidemiologists in many countries have gained almost a celebrity status. A standard practice of crisis communication, frequent live press conferences, has provided a powerful image of “evidence-based” policy. In the spring, some Swedes had the face of a chief epidemiologist, Anders Tegnell, tattooed on their bodies.

At first sight, this celebrated image of the scientist comes in sharp contrast to the way expertise has been troubled by the obstacles of the communicating of the climate crisis: the messaging and lobbying power of industry, the challenge of presenting scientific uncertainty without jeopardizing the larger unassailable findings, or the thorny connections between ideology, identity, and necessary climate action. The climate crisis has not turned scientists into national figure heads, and the idea of ongoing press briefings with climatologist and presidents or prime ministers seems indeed a bit far-fetched.

However, the further the epidemic has spread, the more we have come to witness the tension between politics and expertise. Nowhere has this been so obvious as in the fragmented, polarized, combative—and vulnerable—political-media ecosystem of the United States. Here’s one example among many:

Unveiling details of “Operation Warp Speed,” a name that references a concept popularised by Star Trek and other science fiction, [President] Trump said ( . . . the) “objective is to finish developing and then manufacture and distribute a proven coronavirus vaccine as fast as possible. We’d love to see if we can do it prior to the end of the year.”

Standing just behind him, Anthony Fauci, an infectious diseases expert wearing a face mask, cast his glance down and reached to adjust his tie. Trump did not wear a face mask. (*The Guardian*, May 14, 2020)

You don’t have to be an expert on political semiosis to see the spin: the sci-fi-laced language overselling science for short-term political gain and the hijacking of Fauci’s expertise as theatrical prop to bolster authority. But you also see the polyphony: the face masks as a metaphor for muted experts, the journalist’s professional room of maneuver “only” to report the small gesture of Fauci, letting us read it as a demonstrative signal of an expert taken hostage.

This is a snapshot of what Kenneth Cmiel and John Durham Peters (2020) call the era of “promiscuous knowledge,” where we *no longer expect* anything “*beyond the mix* of the popular and professional, political actor and credentialed expert” (p. 254, my emphasis). At the same time, the scene is symptomatic of a broken public discourse, indeed highlighting the need to think beyond the paradigm of a polyphony vague plurality of “knowledges.” Without a claim for an *epistemic dimension for public discourse*—a capacity to come to terms with the world, and to know what should be done—both journalism and public expertise will wither.

## Transformation of Structures: What Will (Nation) States Come to Mean?

Thinking about systemic crises raises questions about structures. In Logics of History, William Sewell (2005) argues that existing “dislocated structures” always underlie such moments, providing a space for collective action to make a lasting difference. His narrative of the French Revolution proceeds from the unstable conjuncture that saw the King and National Assembly present rival views of power and new ideas about the authority of popular will and, from there, moves to the material, existential crisis of everyday life in which the food supplies left the masses starving and desperate. When Parisians stormed the Bastille in July 1789, they were also helping to forge a new concept to the world—the idea of modern political revolution, in which popular and sometimes even violent revolt can represent legitimate popular sovereignty. It was a lasting transformation of symbolic structures, shaping future agency (Sewell, 2005, pp. 226–270).

The Covid-19 pandemic highlights existing structural fissures in the political order. Most obviously, the virus has entered a context where the twentieth-century commonly held distinction between left and right is being replaced by the polarity that features “nationalists-conservatives” and “cosmopolitan-liberals”—a polarity moving into place long before the coronavirus began moving through the neighborhoods of Wuhan.

Journalism routinely leans toward nationalism, particularly in moments of crises, and the coverage of the pandemic has centered much on the actions taken by national governments to protect national populations. The “nation” shows its dual political power here. On one hand, the nation is depicted as a positive frame of interpretation that can evoke a sense of solidarity and moral responsibility. On the other hand, this frame can also cover up crucial differences and moral discrepancies between nations and among diverse populations within nations. We heard the words “protecting,” “right to life,” and “vulnerable groups” mostly in reference to “our” citizens.

A stronger naturalization of the national grammar of news spells potential problems for transnational experts and other social actors to promote better climate communication. It can easily empower the latest arguments against rapid climate action, arguing that climate change might indeed be real, but it makes little sense for “us” to change our ways—because (for instance) of China’s raising emissions and increasing overpopulation. As such narrow-minded and short-sighted political realism gains strength, the position of journalism addressing the climate crisis weakens.

This is not to recommend more of hollow praise cosmopolitanism and the blessings of globalization. Instead, journalists should be pushed to do work that looks *inside* the nationalistic frames from which they report virus contagion numbers and death tolls—or the emission footprints, drought, flood damage, and species die-offs of the climate change era. At the same time, we need perspective that looks at our nations from the *outside*. These tasks demand well-cultivated transnational professional, expert, and advocacy networks that support the authority of journalism. Covering the pandemic is a key moment for *re-articulating what “nations” and “states” mean in the era of systemic, global crises*. In this sense, Covid-19 coverage plows the soil for future climate coverage.

## A New Horizon: Everyday Life in a Low-Carbon Society

Hannah Arendt’s (1958) The Human Condition argues that events have no causes. Rather, they take place where contingent material *things* intervene with developing human interpretations. In this line of reasoning, it was Galileo’s telescope (rather than the man himself) that pushed the emerging modern world around a corner, giving “an earth-bound creature” the chance to see itself from the perspective of the vast universe. Although the Copernican revolution was already developing as “speculation and imagination,” the telescope turned it into a sense-related, *material observation* (Arendt, 1958, p. 260).

You do not have to be much of a philosopher to see the analogue to the virus. The virus drills another wormhole into the modern bulwark between social and natural order and shows how a material factor becomes an actor in our social and political networks. For the media-Covid19-climate intersection, however, the point is not only to fuel a higher awareness of a global post-human condition.

Perhaps for journalism, the key lesson is more mundane: the acute, material power of the pandemic to change our everyday life. It has forced in a glimpse of a new perspective: how routines of individuals, institutions, and nations *can be redefined* in a systemic crisis. This is the point that Bruno Latour (2020) made recently, calling the pandemic a “dress rehearsal” for climate change. When the background of everyday expectation shifts, what counts as “news” or worthy journalistic storytelling changes too.

Concern about public expertise and worry about legacy of narrow nationalism are important—but they are also drawn from the usual repertoire of critical media scholarship. For better and worse, they point to journalism’s structural alliances that play a powerful role as journalism adjusts to the post-pandemic context. The idea of pandemic as a “dress rehearsal” raises a somewhat new question. Teresa Ashe (2020) formulated that well in a talk in January, just a moment before our horizon changed. How might journalism help us imagine a different kind of *everyday life* that a low-carbon society will demand?
